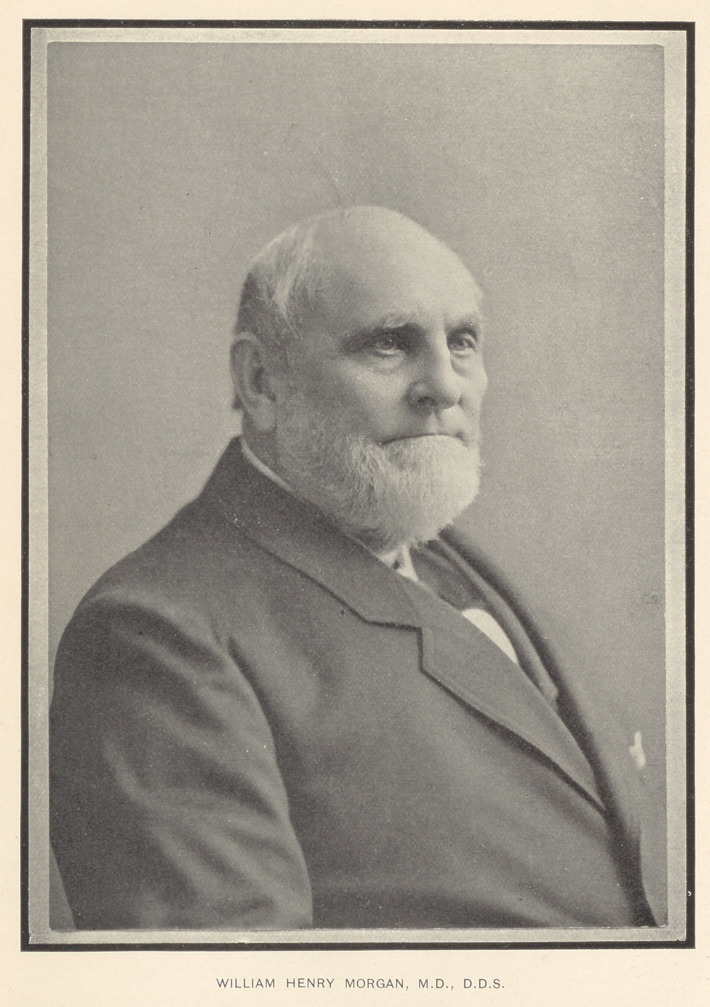# William Henry Morgan, M.D., D.D.S.

**Published:** 1901-07

**Authors:** 


WILLIAM HENRY MORGAN, M.D., D.D.S.

and pressing accurately to place, and then allowing the cement to set in contact with the tissues.

"This refit," the doctor says, "will apply to cases in which a plate has been made soon after extraction, those in which the tissues have become flabby and yielding, as well as those entire dentures in which a leak in the periphery easily admits air beneath the plate."—R. C. Brophy, *The Bur.*

---

### Obituary.

### WILLIAM HENRY MORGAN, M.D., D.D.S.

The death of this distinguished member of the dental profession was briefly noticed in the last number of this journal. His death occurred on Thursday, May 16, 1901, after a long period of failing health, to which was added the feebleness of an advanced age,—eighty-three years.

Dr. Morgan was born in Logan County, Ky., February 23, 1818, and was the son of Joseph and Elizabeth Morgan. His father was a veteran of the war of 1812, having fought under General Jackson at the battle of New Orleans, and his grandfather was a colonel in the Revolutionary War.

The subject of this sketch early established a taste for literary work, and gave a large portion of his scanty earnings on the farm to the purchase of books. His early experiences in farm life gave him but limited opportunities for educational advantages, but his tastes and determined character overcame all obstacles, and he became distinguished in the various positions he was called to fill during his long life.

He graduated from the Baltimore College of Dental Surgery in 1848. He practised dentistry for one year in Russellville, Ky., and then removed to Nashville, Tenn., where he entered into partnership with Dr. T. B. Hamlin. These two were the only practitioners in Tennessee who had, at this time, graduated from a dental college. This partnership continued for ten years.

Dr. Morgan was elected, in 1865, a member of the Board of Trustees of the Ohio College of Dental Surgery, and at a later period was elected its president, which position he resigned in 1879 to accept the chair of Clinical Dentistry and Dental Pathology in Vanderbilt University. The Department of Dentistry of this University was organized by him, and he held the position of dean until his advanced age compelled him to give up the work. Dr. Morgan's reputation extended beyond the confines of his State and neighboring States, and became national. He assisted in organizing the American Dental Association in 1860, and was twice elected to its presidency, the only man at that time thus honored.

He was during a long period a trustee of the Meharry Medical College, and took a deep interest in the education of the colored youth in that institution, especially in the department of Dentistry. He was president, at times, of various dental organizations.

During President Cleveland's administration Dr. Morgan was appointed a member of the Indian Commission, and continued in that service under a portion of President Harrison's administration.

Dr. Morgan was active in religious matters, having early joined the Methodist Church. He was for years superintendent of the Sunday-school connected with his church, and frequently a delegate to the annual Conference and to the General Conference. For thirty years he was a member of the Book Committee of the Methodist Episcopal Church South.

Dr. Morgan ranked high in Freemasonry, being a member of the Scottish Rite, and served for many years as Prelate of the Nashville Commandery, Knights Templar.

While this many-sided life is interesting, as all such lives are, the details that necessarily made it of great value must, even if known, be omitted. Dr. Morgan was true to his own conceptions of duty. He marked out his course of life when very young, and followed it to the end. He had no narrow ambition contracted within religious, political, or State lines, but, grasping the necessities of all classes, he became a strength to the humble and a constant source of inspiration to those more advanced.

His work in his profession was not so much in its literature as it was in practical efforts to influence others through organized effort. As already stated, his ability as a debater and parliamentarian made him efficient in the various positions upon the floor or in the chair. No one was listened to with more respect than he in the discussions upon scientific questions arising in the national body. His mind was a storehouse of facts of observation, upon which he was able to draw to vividly illustrate his subject.

He was not able to be present at the closing meeting of the American Dental Association, which he had so long honored by his presence and co-operation, but his interest was manifested in his messages to his friends.

He was almost the last of that brilliant coterie of men who opened up the last half of the nineteenth century and were active in the renaissance of dentistry at that period. He was contemporary with Harris, Townsend, White, Westcott, Arthur, Taft, Rich, McKellops, and a host of others.

To the writer Dr. Morgan's life and work were ever an inspiriting force. He was to him always a guide in those things that make for progress, and now that he has passed from earthly activities, there seems a void that we may look in vain to fill. In the presence of a life such as this the writer has only the grateful thought that it was his privilege to know and associate with it and to be a partaker of that fine enthusiasm that made Dr. Morgan's life a continual benediction to all who were brought within its influence.

Dr. Morgan was married, in 1852, to Miss Sarah A. Noel, of Kentucky, and was the father of four children,—Mrs. C. H. Noyes, of Warren, Pa., Dr. Henry W. Morgan, J. B. Morgan, and Garrett Morgan.

The funeral took place at the McKendree Church, May 17; interment in Mount Olivet Cemetery, Nashville.

## Figures and Tables

**Figure f1:**